# The Slaughter of the Innocents

**Published:** 1872-12

**Authors:** 


### THE SLAUGHTER OF THE INNOCENTS.

The "Evening Star," of Washington City, makes the following comments upon the "vital statistics" prepared by Dr. J. M. Toner, of that city. We are sure our readers will read the facts presented with very great interest:

" Attention is frequently called to the mortality among children in particular seasons or particular localities, but few seem to be aware of the frightfully large percentage of infant mortality year by year, compared with the whole number of deaths of all ages. The mortality tables prepared by Dr. J. M. Toner, of this city, whose careful and intelligent labors in gathering the vital statistics of the country, have been referred to heretofore, present some startling facts in this connection. These tables show the annual mortality in several of the States, and in a number of our large cities, made up from their published reports for the years named, giving the whole number of deaths, and also the number dying under five years of age; the number dying between five and ten years; also, the number dying from cholera infantum, with the per cent. of each class.

The census of the United States for 1850 showed a per centage of 39.76 of deaths of children under 5 years of age to the total deaths of all ages; and the per centage in the census of 1860 had increased to 43.15. The mortality under 5 in the great cities of the country has risen to a much higher rate, the highest being in Chicago, where it has mounted from a per centage of 28.67 in 1843, to 62.83 in 1869. In 1868 the number of deaths of children under 5 to the to the total deaths of all ages stood in that city in the frightful proportion of 3,713 of the former to 5,984 of the latter, and in 1869 it had risen to 4,077 under 5 to 6,488 over that age.

Next comes St. Louis, where, in 1871, the per centage was 51.10; the number of deaths under 5 being 3,409 to 6,670 above.

Ranging about the same as St. Louis is the per centage of infant mortality in New York. In New York city in 1869 the per centage was 51.09; the number of deaths under 5 in that year being 12,359 to 25,167 above. In that city it would appear, however, that the proportion of deaths under 5 is rather diminishing than otherwise, as during the series of years from 1835 to 1853 it had ranged from 51.03 to 57.10, while in 1866 it was but 47.73, in 1867 it was 52.10, and in 1869 but 51.09. In the year 1811 the mortality in New York amongst the children seems to have been exceptionally large, as the per centage was 77.09; the number of deaths under 5 being 1,946 to 2,524 above that age.

In Philadelphia, too, the chances of the little folks in the fight for life seems to be rather improving, as the per centage of deaths under 5 stood as high as 51.87 in 1853 and 51.20 in 1861, while from 1862 to 1869 it has ranged from 43.22 to 46.63, and in 1870 was but 44.27.

Next to New York in infant mortality comes Baltimore with its per centage, in 1869 of 49.85 under 5 years, though the proportion has increased but little since 1860, when it stood 48.74.

Cincinnati, in 1868, had a per cent. of 46.68 under five; and Philadelphia, as above stated, the next highest per centage. In New Orleans, the returns are brought down only to 1857, when the per centage was 43.85. Providence, Rhode Island, seems to be a favorable place for the little ones, as the mortality under five has not, from 1865 to 1870, been above 36.99.

In this city, the mortality returns have been so inadequately made, that only scattered statistics concerning separate years can be obtained. In 1849, the number of deaths under five was 400, to 866 above—a per centage of 46.18. In 1862, there were 547 deaths under five, to 1,115 above—a per centage of 49.05. In 1858, the per centage was 45.78, the number of deaths under five being 429, to 437 above.

The mortality statistics of Richmond present some curious features, as showing that while the number of deaths of colored children under five is nearly two to one that of the whites of the same age, the proportion of white children dying of cholera infantum is nearly double the colored mortality from the same disease, there being 490 white deaths in 1869 of cholera infantum to 250 colored in the same year in that city.

In view of the appalling proportion of infant mortality, especially in our large cities, shown by the statistics above referred to, Dr. Toner some time ago called attention to the necessity for adopting some general measures for checking this vast waste of infant life. We have various humane institutions, insane asylums, etc., to alleviate the sufferings and lengthen out the lives of the wrecks of humanity, but we have literally nothing to stay the immense waste of life of the hopeful young, full of great promise. It is certainly better to provide for the preservation of the lives of those who will grow into usefulness, than to confine all our humane efforts to guarding the health of the hopelessly shattered wrecks.

Of course, wealthy parents have it in their power to remove their ailing children to purer air, but it is an unpleasant task for a physician to say to parents, unable to meet the expense, that the life of a child suffering from cholera infantum depends on its being removed to another locality. To meet this difficulty, Dr. Toner suggests the establishment of free parks at elevated points, where parents, or mothers and nurses, may camp out in the summer months in such style as suits their means or convenience, either in tents or cheap cottages. Cities and communities should feel it a moral duty to aid in any measure of the kind for the preservation of infantile life. Cheap fare, or free fare, should be furnished to poor women with their sick babies, and they should be aided with rations on the ground. Philanthropic people, no doubt, would willingly aid, if the means were pointed out, in some systematic measure of relief for the suffering little ones who perish by the thousands yearly in our large cities for want of air and attention. The child in such cases is not sick, but is suffering simply from excess of heat. There is no solid tissue in the infant. It is virtually all liquid; it gets heated up, and deterioration takes place. What is wanted is to keep down the temperature. Infants do not perspire as adults do, but the process of retained heat goes on with them, and they suffer accordingly. Evaporation should be encouraged; they should be sponged frequently, and exposed to the air as much as possible; and, above all, there should be no flannels next to the skin in hot weather. Many mothers really destroy all chances their ailing babies have for life by bandaging them with flannels, and sedulously keeping them out of the fresh air lest they should take cold in a draught. Infants, for the reasons above explained, can hardly have too mnch air.

---

BOOKS.—We have on hand several books, which will be noticed in the January number.

